# Deletion of epidermal Rac1 inhibits HPV-8 induced skin papilloma formation and facilitates HPV-8- and UV-light induced skin carcinogenesis

**DOI:** 10.18632/oncotarget.11069

**Published:** 2016-08-05

**Authors:** Jayesh Deshmukh, Ruth Pofahl, Herbert Pfister, Ingo Haase

**Affiliations:** ^1^ Department of Dermatology, University of Cologne, Cologne, 50937, Germany; ^2^ Institute of Virology, University of Cologne, Cologne, 50935, Germany

**Keywords:** HPV-8, Rac1, skin cancer, SCC, UV (Ultraviolet) light

## Abstract

Overexpression and increased activity of the small Rho GTPase Rac1 has been linked to squamous cell carcinoma of the epidermis and mucosa in humans. Targeted deletion of Rac1 or inhibition of Rac1 activity in epidermal keratinocytes reduced papilloma formation in a chemical skin carcinogenesis mouse model. However, a potential role of Rac1 in HPV- and UV-light induced skin carcinogenesis has not been investigated so far, solar UV radiation being an important carcinogen to the skin.

To investigate this, we deleted Rac1 or modulated its activity in mice with transgenic expression of Human papilloma virus type-8 (HPV-8) in epidermal keratinocytes. Our data show that inhibition or deletion of Rac1 results in reduced papilloma formation upon UV-irradiation with a single dose, whereas constitutive activation of Rac1 strongly increases papilloma frequency in these mice. Surprisingly, we observed that, upon chronic UV-irradiation, the majority of mice with transgenic expression of HPV-8 and epidermis specific Rac1 deletion developed squamous cell carcinomas. Taken together, our data show that Rac1 exerts a dual role in skin carcinogenesis: its activation is, on one hand, required for HPV-8- and UV-light induced papilloma formation but, on the other, suppresses the development of squamous cell carcinomas.

## INTRODUCTION

Non-melanoma skin cancer (NMSC), which includes basal cell carcinoma and squamous cell carcinoma (SCC), is the most common skin cancer in the fair skinned population [1]. Approximately 5 million new cases of NMSC are reported every year worldwide [2], usually in sun-exposed areas of the human body. Solar ultraviolet (UV) radiation has a strong epidemiological association with the development of skin cancer and is considered as an important carcinogen [3]. In addition, beta-papillomavirus infection of epidermal keratinocytes is thought to be an important etiological factor for the development of SCCs [4–6]. Human papilloma viruses of the Beta genus, especially HPV-5 and HPV-8, are thought to be able to cause SCCs in cooperation with UV-light [6]. Beta genus HPVs are commonly found in lesions of patients with the rare disease epidermodysplasia verruciformis (EV) [7]. EV patients are highly susceptible for beta HPV infections and often develop SCCs of the skin.

Mouse models have been widely used for the investigation of chemically induced, HPV-8 induced as well as UV-light induced skin carcinogenesis. In these mouse models early appearance of benign papillomas is very common which then rarely progress to invasive SCCs. In humans, SCCs usually develop without preceding papillomas; skin papillomas are mostly associated with viral infections and rarely caused by chemical carcinogens or UV radiation [8, 9].

Rac1 is a small Rho family GTPase from the Ras superfamily. Like the majority of other Rho GTPases, Rac1 switches between its GDP bound inactive form and GTP bound active form. Various mediators can regulate the transition of Rac1 between these two stages. Guanine nucleotide exchange factors (GEFs) such as Tiam1, Vav2 and Trio catalyze the exchange of GTP for GDP to activate Rac1. After activation, Rac1 interacts with various effector molecules coordinating downstream signaling pathways [10].

Rac1 is over-expressed in various human cancers such as testicular, gastric and breast cancers as well as oral SCCs [11]. Over-activation of Rac1 was also observed in SCC samples of human skin and in cell lines derived from head and neck SCCs [12, 13]. Results of experiments in several mouse models indeed support a potential role of Rac1 as an oncogene. In a colorectal carcinoma model, Rac1 deletion decreased tumor progression while over-expression of Rac1 promoted tumor formation [14]. Targeted deletion of Rac1 or inhibition of Rac1 activity by the chemical inhibitor NSC23766 in epidermal keratinocytes reduced skin tumor formation in a chemical skin carcinogenesis model [12, 15].

A potential role of Rac1 in HPV-8 and UV-light induced skin carcinogenesis has not been investigated so far. To study this, we deleted Rac1 or modulated its activity in mice with transgenic expression of the complete early region of HPV-8 in epidermal keratinocytes [16]. We show that, on one hand and in agreement with previous studies, inhibition or deletion of Rac1 results in a reduced number of spontaneous or UV-light induced papillomas in K14 HPV-8 mice, whereas constitutive activation of Rac1 facilitates papilloma formation in these mice. On the other hand, we observed, to our surprise, that the majority of mice with epidermis specific Rac1 deletion developed SCCs upon chronic UV-irradiation. We thus present direct evidence for a so far unknown dual role of Rac1 in chronic UV-light induced skin carcinogenesis: Rac1 is required for the formation of skin papillomas but its absence facilitates the formation of SCCs in K14 HPV-8 mice.

## RESULTS

All experiments were approved by the review board of the government of Northrhine Westfalia, Germany. Comparisons of phenotypes were only made within the same mouse strain in all the experiments.

### Inhibition or deletion of Rac1 in the epidermis prevents spontaneous HPV- 8 induced skin papilloma formation

**Figure 1:**
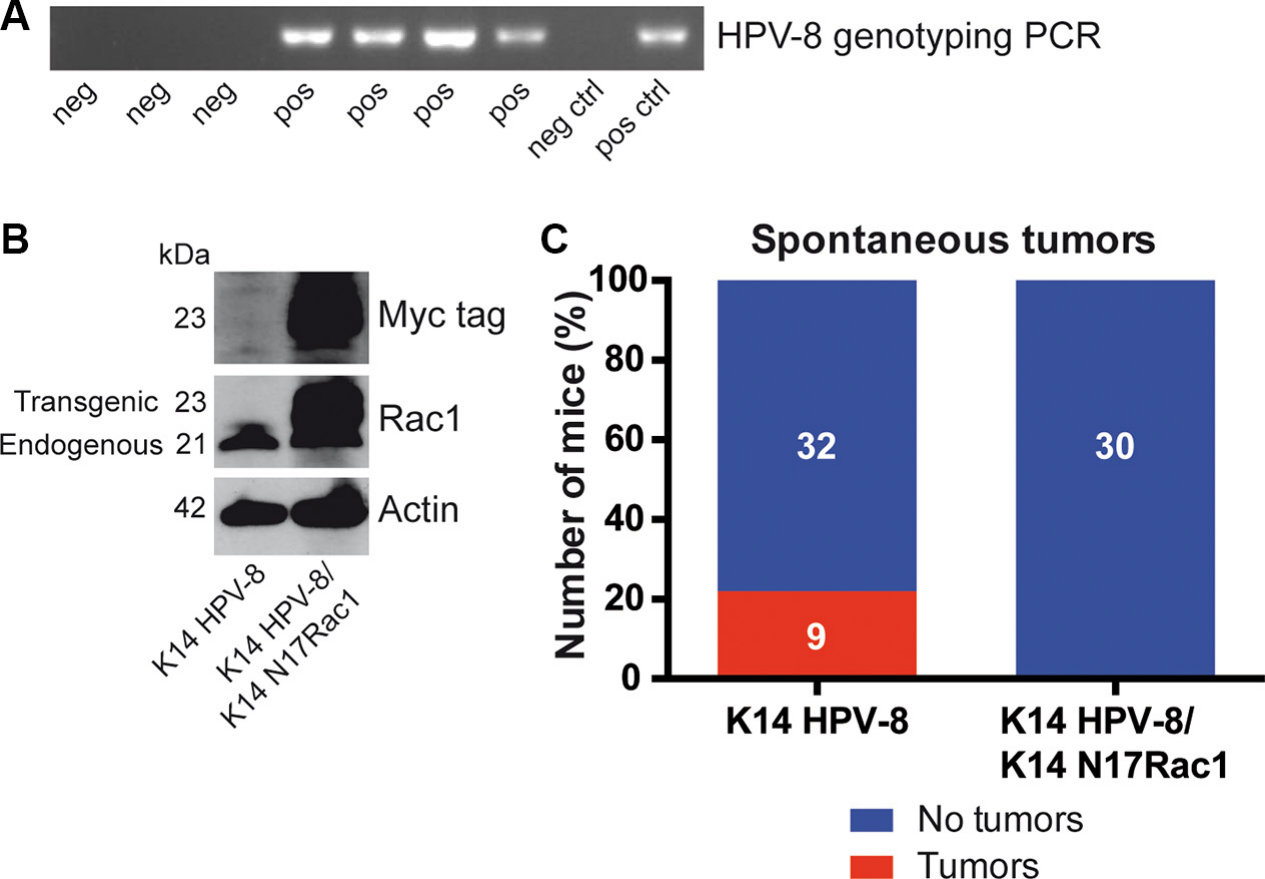
Epidermis specific inhibition of Rac1 inhibits HPV-8 associated spontaneous papilloma formation (**A**) PCR genotyping of HPV-8 transgenic mice and controls. (**B**) Western blot analysis of myc-tag and Rac1 expression of cell lysates from K14 HPV-8 and K14 HPV-8/K14 N17Rac1 transgenic keratinocytes. Numbers on the left indicate molecular weights in kDa. (**C**) Graph shows the percentage of K14 HPV-8 and K14 HPV-8/K14 N17Rac1 mice with (red) and without (blue) spontaneously developing skin tumors. Absolute numbers of mice are shown within the bars.

Rac1 has been reported to be essential for skin papilloma formation in chemical skin carcinogenesis experiments [15]. Mice expressing the complete early region of HPV-8 under the control of the keratin 14 (K14) promoter show spontaneous skin papilloma development from the age of 14 weeks [16] and therefore provide a unique model to study HPV-8 induced papilloma formation (Table 1, Figure 1A). To investigate whether the activity of Rac1 is required for HPV-8 induced papilloma formation, we compared the spontaneous occurrence of skin papillomas in K14 HPV-8 transgenic mice and mice expressing a myc tagged inhibitory mutant of Rac1, K14 N17Rac1, in addition to HPV-8, both in the basal compartment of the epidermis (K14 HPV-8/K14 N17Rac1) (Table 1, Figure 1B) [17]. In 9 out of 41 (22%) K14 HPV-8 mice we observed spontaneous papilloma development whereas none of the K14 HPV-8/K14 N17Rac1 mice developed spontaneous papillomas (Figure 1C).

**Table 1:** Mouse models used in this study

Mouse models	Genotypes	Background	References
K14 HPV-8*	K14 HPV-8	C57/Bl6	[16]
**K14 HPV-8/K14 N17Rac1**	K14 HPV-8; K14 N17Rac1	C57/Bl6	[16, 17]
**K14 HPV-8**	K14 HPV-8	FVB/N	[16]
**K14 L61Rac1**	K14 L61Rac1	FVB/N	[19]
**K14 HPV-8/ K14 L61Rac1**	K14 HPV-8; K14 L61Rac1	FVB/N	[16, 19]
**K14 HPV-8/ Rac1-EKO**	K14 HPV-8; K14 Cre; Rac1 fl/fl	FVB/N	[16–18]
**K14 HPV-8/ Rac1-EKO^het^**	K14 HPV-8; K14 Cre; Rac1 fl/+	FVB/N	[16–18]

* K14 HPV-8 mice in C57/Bl6 background were used as controls with K14 HPV-8/K14 N17Rac1 mice in C57/Bl6 background. All other mice were used in FVB/N background. K14 HPV-8: complete early region of HPV-8 expressed under the control of K14 promoter. K14 N17Rac1: Dominant negative mutant of Rac1 expressed under the control of K14 promoter. K14 L61Rac1: Constitutively active mutant of Rac1 expressed under the control of K14 promoter. Rac1-EKO and Rac1-EKO^het^: Cre recombinase expressed under the control of K14 promoter with two floxed alleles of Rac1 (Rac1-EKO) or one floxed allele and one wild type allele (Rac1-EKO^het^).

**Figure 2:**
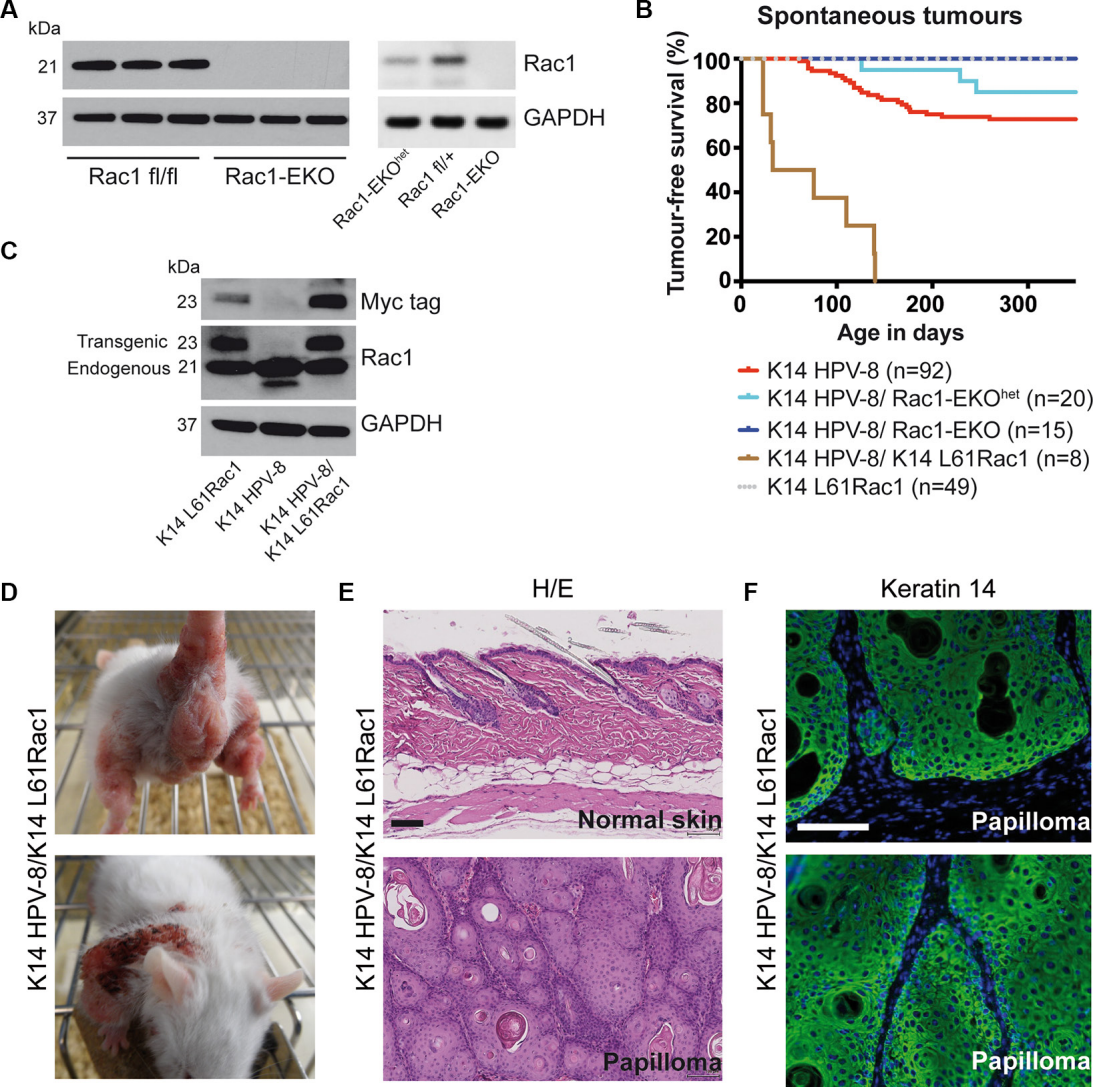
Epidermis specific deletion or activation of Rac1 determines HPV-8 associated spontaneous papilloma formation (**A**) Western blot analysis of Rac1 expression of keratinocyte lysates from Rac1 fl/fl, Rac1 fl/+, Rac1-EKO^het^ and Rac1-EKO keratinocytes. Rac1 fl/fl and Rac1 fl/+ do not express K14 Cre. GAPDH and actin are used as loading controls. Numbers on the left indicate molecular weights in kDa. (**B**) Kaplan-Meier curve of tumor free survival of spontaneous tumor development in groups of mice with indicated genotypes over time. K14 HPV-8/Rac1-EKO and K14 L61Rac1 mice did not show any spontaneous tumors. (**C**) Western blot analysis of myc-tag and Rac1 expression of whole skin lysates from K14 L61Rac1, K14 HPV-8 and K14 HPV-8/K14 L61Rac1 transgenic mice. (**D**) Representative photographs of K14 HPV-8/K14 L61Rac1 mice with spontaneous tumors (**E**) H/E staining of skin sections of normal skin (upper panel) and a skin papilloma (lower panel) of K14 HPV-8/K14 L61Rac1 mice. (**F**) Keratin 14 immunostaining (green) of a skin papilloma of a K14 HPV-8/K14 L61Rac1 mouse. Nuclei are stained in blue. Scale bar = 100 μm.

In an alternative approach, we deleted Rac1 specifically in the epidermis of K14 HPV-8 mice (K14 HPV-8/Rac1-EKO) [18] (Figure 2A). We followed these K14 HPV-8/Rac1-EKO mice and K14 HPV-8 mice as controls over 350 days and recorded the appearance of skin papillomas. We observed spontaneous papilloma formation in 25 out of 92 (27.2%) of K14 HPV-8 mice, starting from post-natal day 61 (P61) (Figure 2B). In contrast, none of the 15 K14 HPV-8/Rac1-EKO mice developed papillomas. Interestingly, only 3 out of 20 (15%) K14 HPV-8 transgenic mice with epidermis specific expression of only one Rac1 allele (K14 HPV-8/Rac1-EKO^het^) developed skin papillomas starting from P126, suggesting a dose effect of Rac1 expression on papilloma formation (Figure 2A right panel and 2B). These results suggest an essential function for Rac1 in the spontaneous development of skin papillomas in K14 HPV-8 mice.

### Activation of Rac1 facilitates papilloma formation in K14 HPV-8 mice

To further analyze a possible co-operation of Rac1 and HPV-8 in skin papilloma formation, we generated double transgenic K14 HPV-8/K14 L61Rac1 mice expressing HPV-8 and, in addition, an activating mutant of Rac1, L61Rac1 [19], both under the control of the K14 promoter (Table 1, Figure 2C). Basal epidermal keratinocytes of these mice express the HPV-8 complete early region and a constitutively active mutant of Rac1 simultaneously. All of these K14 HPV-8/K14 L61Rac1 double transgenic mice (8 out of 8) developed skin papillomas by P140 (Figure 2B, 2D), while only 18% of the K14 HPV-8 mice without expression of L61Rac1 developed papillomas and none of the K14 L61Rac1 mice developed papillomas.

All spontaneous tumors from K14 HPV-8 mice, K14 HPV-8/Rac1-EKO^het^, K14 HPV-8/K14 N17Rac1 and K14 HPV-8/K14 L61Rac1 mice were analyzed histologically. H&E staining and K14 immunostaining revealed acanthosis as well as hyper- and parakeratosis but clear demarcation from the underlying dermis without or with very mild cellular atypia, clearly showing that these tumors were papillomas, not squamous cell carcinomas (Figure 2E, 2F).

These data show that inhibition or the absence of Rac1 decreases or prevents skin papilloma formation, whereas constitutive activation of Rac1 strongly increases the development of skin papillomas in K14 HPV-8 mice.

### Inhibition of Rac1 inhibits UV-light induced skin papillomas in K14 HPV-8 mice

**Figure 3:**
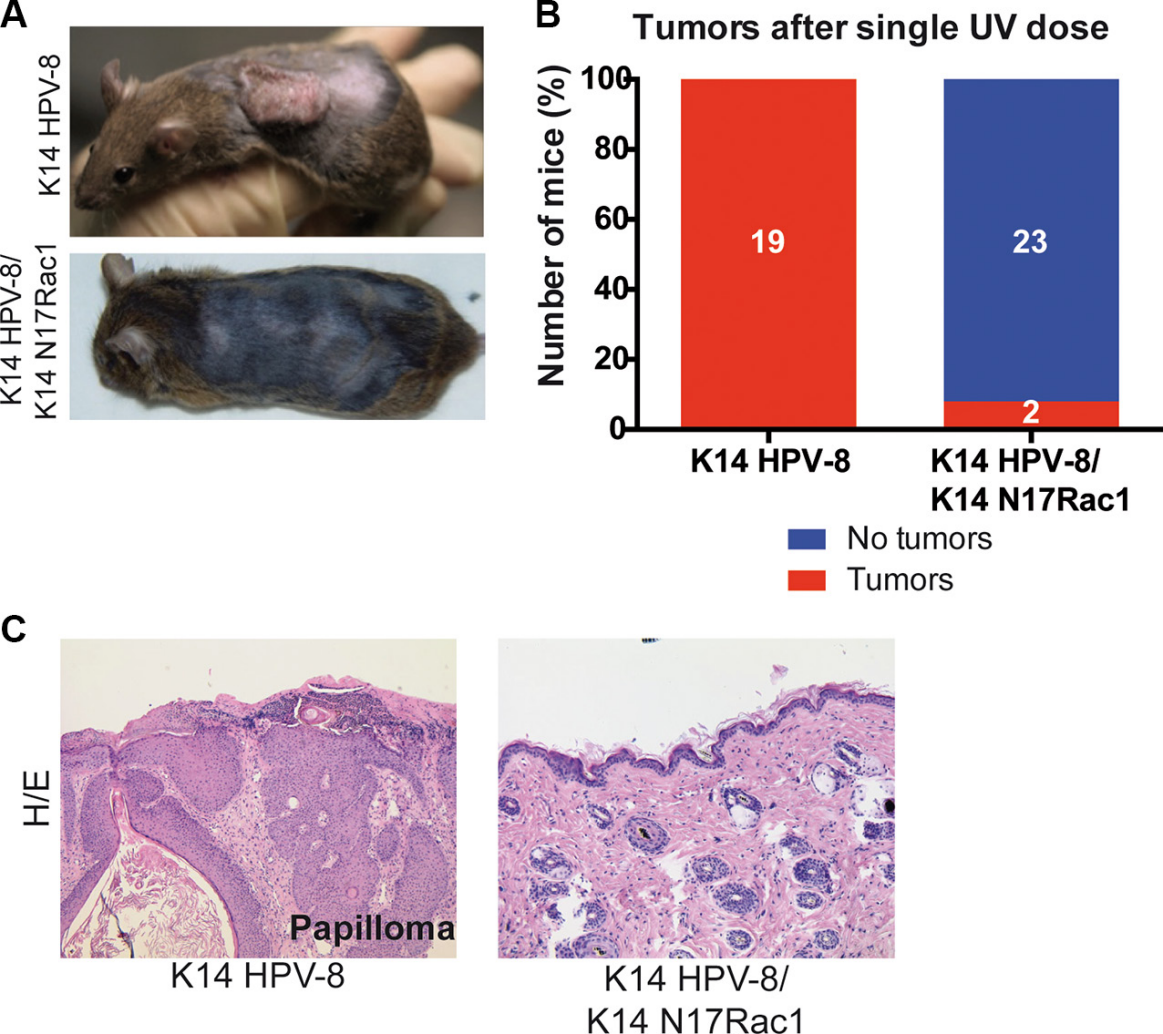
Epidermis specific inhibition of Rac1 attenuates UV-light induced skin tumor formation in K14 HPV-8 mice (**A**) Representative photographs of K14 HPV-8 and K14 HPV-8/K14 N17Rac1 mice about 3 months after one single dose of UV-irradiation. (**B**) Graph shows the percentage of K14 HPV-8 and K14 HPV-8/K14 N17Rac1 mice with (red) and without (blue) spontaneously developing skin tumors. Absolute numbers of mice are shown within the bars. (**C**) H/E staining of skin sections of UV-light induced skin papilloma from K14 HPV-8 mice and UV-irradiated skin of K14 HPV-8/K14 N17Rac1 mice.

Human papilloma viruses of the Beta genus, such as HPV-8, are thought to be able to cause SCCs in cooperation with UV-light [6]. Since, for human skin, the most relevant environmental carcinogen is UV-light [3], we asked whether Rac1 activity plays a similar role in UV-light induced skin tumor formation. In K14 HPV-8 transgenic mice, skin papilloma formation is induced by a single irradiation with UV-light [20]. We therefore compared skin papilloma formation in K14 HPV-8 mice and K14 HPV-8/K14 N17Rac1 mice after a single dose of UVA (10 J/cm^2^) and UVB (1 J/cm^2^). Until 12 weeks after irradiation, all K14 HPV-8 mice (19 out of 19) developed visible skin papillomas (Figure 3A–3C). In contrast, only 2 out of 25 (8%) K14 HPV-8/K14 N17Rac1 mice developed small papillomas, whereas 23 of these mice did not show macroscopic or microscopic signs of skin tumors (Figure 3A–3C). Similar to spontaneous papillomas, UV-light induced papillomas in K14 HPV-8/K14 N17Rac1 mice did not show histological signs of malignancy (Figure 3C). These results show that the presence of the inhibitory mutant of Rac1, N17Rac1, strongly inhibits UV-light induced skin papilloma formation in K14 HPV-8 mice.

### Deletion of Rac1 in the epidermis of K14 HPV-8 mice results in the development of SCCs upon chronic UV-irradiation

**Figure 4:**
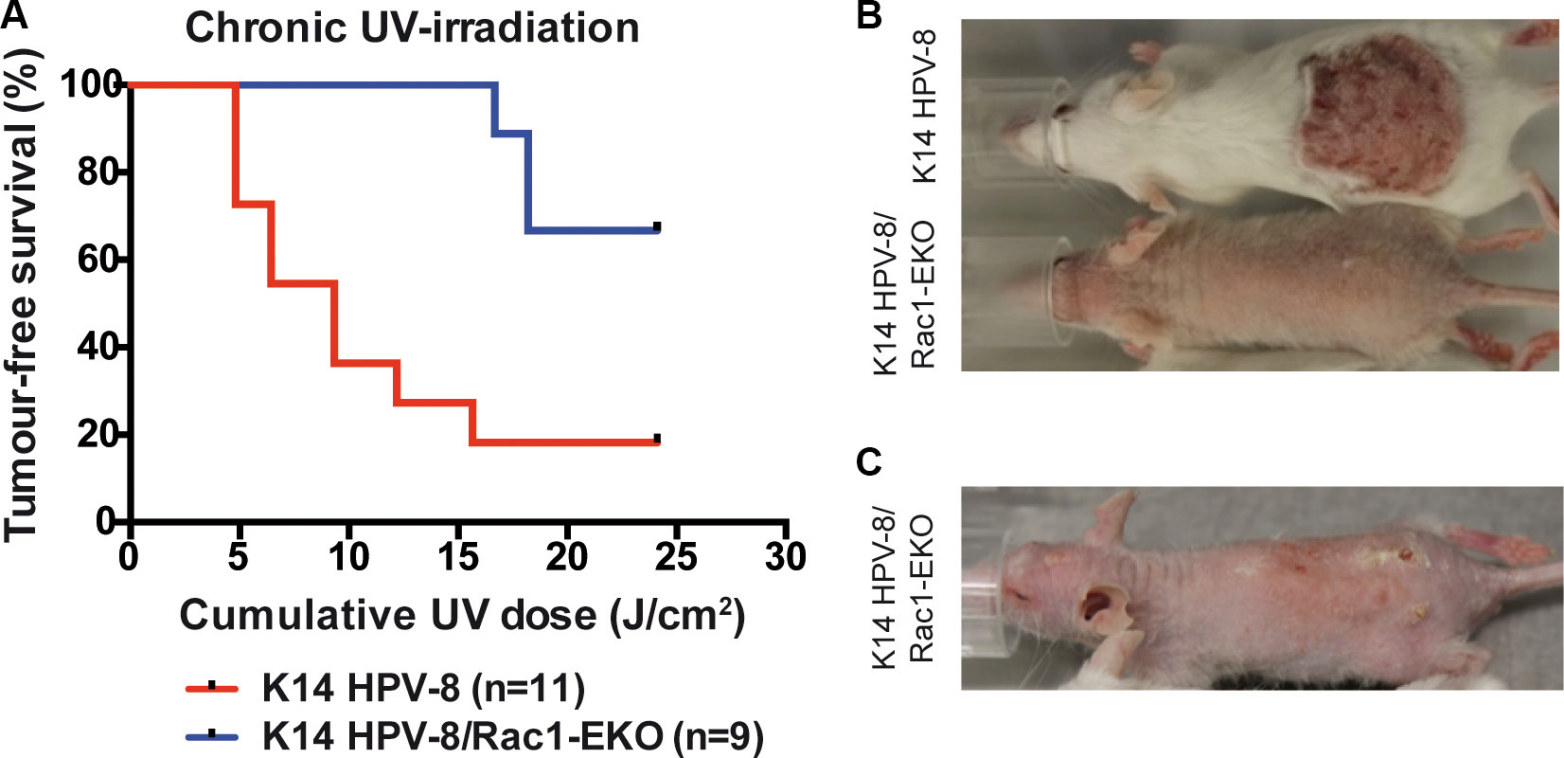
Epidermis specific deletion of Rac1 attenuates chronic UV-light induced papilloma formation in K14 HPV-8 mice (**A**) Tumor free survival of K14 HPV-8- and K14 HPV-8/Rac1-EKO mice with increasing cumulative dose of UV-light. (**B**, **C**) Representative photographs of K14 HPV-8 and K14 HPV-8/Rac1-EKO mice treated with chronic UV-irradiation at a cumulative dose of 6.4 J/cm2 (B) and K14 HPV-8/Rac1-EKO mouse treated with chronic UV-irradiation at a cumulative dose of 18.21 J/cm2 (C).

**Figure 5:**
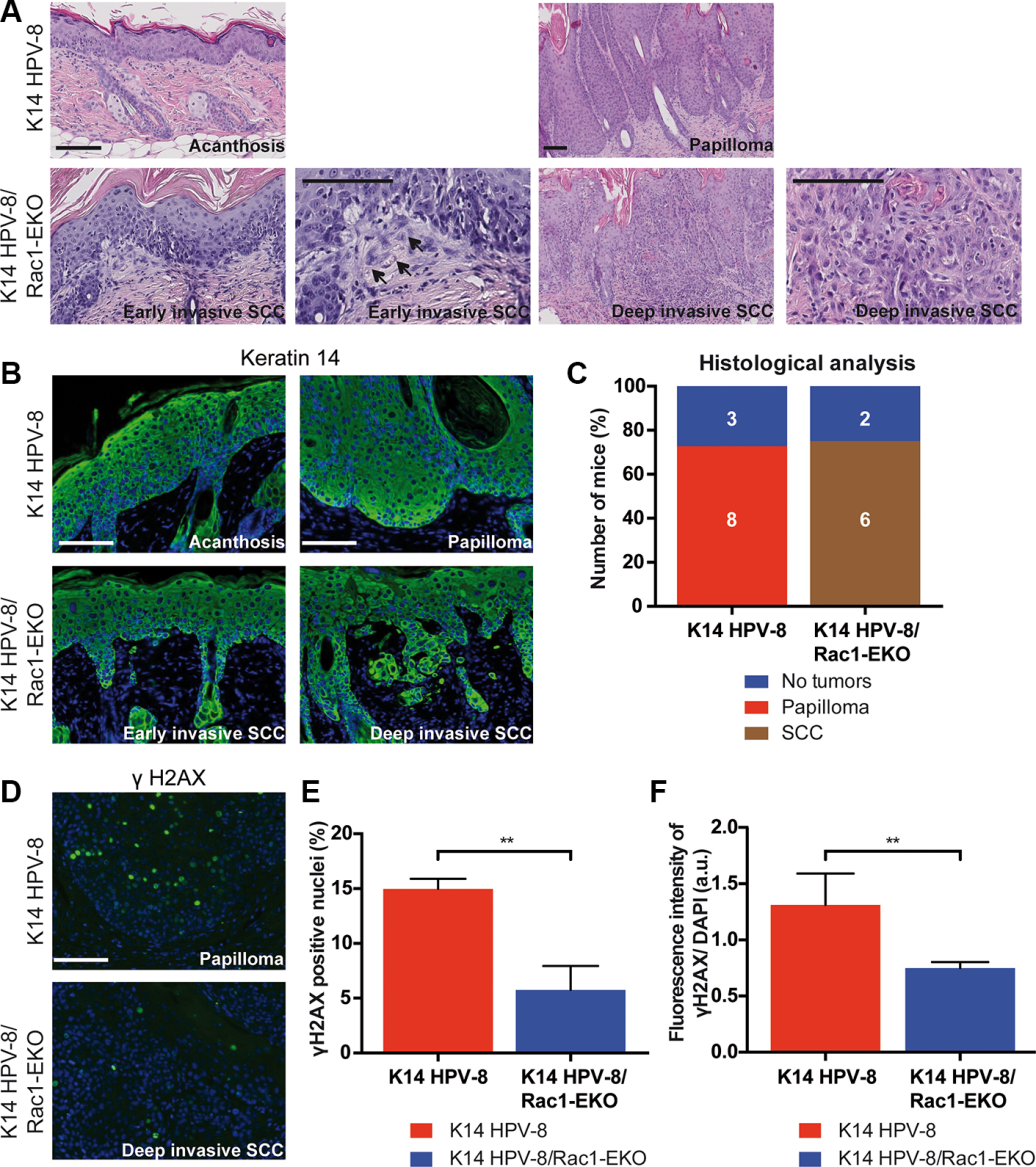
Epidermis specific deletion of Rac1 leads to SCC formation in HPV-8 mice (**A**, **B**) H/E staining (A) and immunostainings against keratin 14 (green) (B) of skin and tumor samples of K14 HPV-8 and K14 HPV-8/Rac1-EKO mice after chronic UV-irradiation. Arrows indicate early invasive changes in K14 HPV-8/Rac1-EKO mice. (**C**) Results of histological analysis of skin tumors from K14 HPV-8 and K14 HPV-8/Rac1-EKO mice. Mice without tumors are shown in blue, mice with papillomas in red and mice with SCCs in brown. Absolute numbers of mice are given within the bars. Scale bar = 100 μm. (**D**) Immunostainings against γH2AX (green) from tumor samples of K14 HPV-8 and K14 HPV-8/Rac1-EKO mice after chronic UV-irradiation. (**E**, **F**) Graphs show percentage number of γH2AX positive cells (E) and fluorescence intensities in arbitrary units (F) from tumor samples of K14 HPV-8 (red bars) and K14 HPV-8/Rac1-EKO (blue bars) mice. Error bars show standard deviation. **represent *p* Value < 0.01

In men, a high cumulative dose of UV-light is believed to be the major cause of age associated skin cancer [2]. We therefore asked whether the presence of Rac1 would also be required for skin papilloma formation in a chronic UV-irradiation protocol in K14 HPV-8 mice [21]. We irradiated 11 K14 HPV-8 mice and 9 K14 HPV-8/Rac1-EKO mice with increasing doses of UV-light. We observed that 9 out of 11 (81.8%) K14 HPV-8 mice developed visible skin tumors, starting from a cumulative UV-light dose of approximately 5 J/cm^2^, whereas only 3 out of 9 (33.3%) K14 HPV-8/Rac1-EKO mice developed clinically apparent tumors starting from a cumulative UV-light dose of approximately 15 J/cm^2^ (Figure 4A–4C). Skin tumors in K14 HPV-8/Rac1-EKO mice were more flat than those in K14 HPV-8 mice (Figure 4C). In agreement with previous results, histological examination revealed that all tumors, which developed in K14 HPV-8 mice, were papillomas (Figure 5A, 5B). In contrast, histological examination of UV-irradiated K14 HPV-8/Rac1-EKO skin revealed a variable degree of acanthosis but also clear signs of malignancy in 6 out of 8 (75%) mice: asymmetric and invasive growth, cellular and nuclear pleomorphism, disturbed differentiation and increased mitotic rate. 4 out of these 6 mice showed deeply penetrating and 2 showed early invasive SCCs (Figure 5A–5C).

UV-light is known to cause DNA damage, which elicits a DNA damage response in epidermal keratinocytes. Recruitment of γH2AX to the site of damage is a commonly used marker of the DNA damage response. To investigate this in K14 HPV-8 and K14 HPV-8/Rac1-EKO mice, we carried out immunostainings for γH2AX on tumor samples. Quantification of γH2AX positive nuclei showed that papillomas in K14 HPV-8 mice (*n* = 3) contain about 3 times as many positive nuclei compared to SCCs of K14 HPV-8/Rac1-EKO mice (*n* = 4) (Figure 5D, 5E). A similar result was obtained when we measured the fluorescence intensities in the same set of samples (Figure 5F).

These results show that the presence and the activity of Rac1 promote chronic UV-light induced papilloma formation in K14 HPV-8 transgenic mice. Furthermore, the presence of Rac1 is required to suppress the development of UV-light and HPV-8 induced squamous cell carcinomas.

## DISCUSSION

Both Rac1 and HPV of the Beta Genus, such as HPV-8, have been implicated in skin carcinogenesis. While HPV-8 was found to be involved in the development of SCCs in sun exposed skin of patients with epidermodysplasia verruciformis, over-expression and activation of Rac1 have been reported in SCCs of mucosa and cornified skin [6, 12, 13]. In our study we identified, in addition, a role of Rac1 in the development of HPV-8 related skin papillomas in mice: inhibition of Rac1 activity or epidermis specific deletion of Rac1 led to a strong decrease in both spontaneous and UV-light induced skin papilloma formation. These results are in line with those of Wang et al. 2010, who found that deletion of Rac1 in the epidermis inhibits chemically induced formation of skin papillomas which are thought to be precursors of SCCs in mice [9, 15]. On the other hand, constitutive activation of Rac1, in addition to expression of HPV-8, led to the development of papillomas, but not SCCs, in 100% of the double transgenic K14 HPV-8/K14 L61Rac1 mice, whereas K14 L61Rac1 mice without HPV-8 did not develop skin papillomas. This shows that Rac1 activity essentially supports HPV-8 induced papilloma formation in skin but cannot induce papillomas by itself. In agreement with the described role of Tiam1 [22], one function of Rac1 may be to transmit growth factor signals from the tumor environment which stimulate papilloma growth. Upon inhibition or in the absence of Rac1, such signals could be ameliorated, which results in a lower number of clinically apparent papillomas.

In K14 HPV-8 transgenics, 6% of the mice that develop papillomas, show spontaneous progression to SCCs [16], indicating a low transforming potential of transgenic HPV-8. Previous studies suggested that the E6 protein of HPV-8 interferes with the repair of UV light induced CPDs in mice and thereby facilitates papilloma formation [23]. In our K14 HPV-8 mice we did not observe progression of papillomas to SCCs, probably due to the limited duration of the observation period. Remarkably, constitutive activation of Rac1 in K14 HPV-8/K14 L61Rac1 transgenic mice did not result in an increased frequency of SCCs, suggesting that the role of Rac1 in skin carcinogenesis is primarily growth promoting and not transforming. In accordance with the results in K14 HPV-8/K14 N17Rac1 mice, K14 HPV-8 mice with epidermis specific deletion developed less skin papillomas, both spontaneously and upon UV-irradiation. To our surprise, however, despite of reduced papilloma formation, the skin of K14 HPV-8/Rac1-EKO mice showed changes with clear features of squamous cell carcinomas. Histological analysis revealed cellular changes typical for squamous cell carcinomas of the skin and showed deep invasive growth in most cases.

How can the absence of Rac1 promote the development of squamous cell carcinomas? Together with integrins and PAR family proteins Rac1 is part of the cell polarizing machinery [24]. Previously, it has been shown that the Rac1 activator Tiam1 regulates the Par3 polarity complex in keratinocytes [25]. In DMBA/TPA chemical carcinogenesis experiments, Tiam1 deficient mice showed a reduced number of papillomas but increased numbers of SCCs [22]. Likewise, epidermal loss of Par3 resulted in a reduced number of papillomas and an increased number of keratoacanthomas, which are highly differentiated squamous cell carcinomas, in chemical carcinogenesis experiments [26]. In addition, it has recently been shown that loss of function mutations in the cell polarity regulator Par3 promote malignant invasion in lung squamous cell carcinomas [27]. It is therefore well conceivable that disruption of Rac1 function interferes with the regulation of keratinocyte polarity in the epidermis of K14 HPV-8/Rac1-EKO mice, thereby promoting the development of SCCs. Reduced strength of cell-cell-adhesion, which is known to be mediated by Rac1, could essentially contribute to this [25]. Such a scenario would be well compatible with our model, since we repeatedly observed single or small groups of epidermal keratinocytes detaching from the epidermis in early invasive SCCs in K14 HPV-8/Rac1-EKO mice (Figure 5A, 5B). Therefore, it is likely that the known role of Rac1 in the maintenance of cell polarity could be an important element of its tumor suppressing function [25].

UV irradiation causes damage of genomic DNA and elicits a cellular DNA damage response. This can result in repair of the damage or withdrawal from the cell cycle, e.g. by senescence. In MCF-7 breast cancer cells, inhibition of Rac1 attenuates the ionizing radiation (IR) induced G2/M arrest and inhibits the DNA damage response pathway [28]. A similar role of Rac1 has also been shown for the IR induced and UV-light induced DNA damage response and repair pathway in HeLa cells [29]. It is therefore conceivable that an altered DNA damage response and repair upon deletion of Rac1 in the epidermis of HPV-8 mice could be responsible for the development of UV-irradiation induced SCCs. The observed difference in the number of γH2AX positive cells in the tumor samples between different mouse groups (Figure 5D–5F) may indicate an inhibited DNA damage response in the skin of K14 HPV-8/Rac1-EKO mice. Interestingly, the E6 oncogene of HPV-8 has been reported to interfere with DNA damage repair [23]. It therefore seems possible that deletion of Rac1 and transgenic expression of HPV-8 E6 could suppress DNA damage repair in an additive way, which would explain why SCCs in K14 HPV-8 mice develop only in the absence of Rac1.

Rac1 has also been implicated in the regulation of cellular senescence [30, 31]. Our stainings of γH2AX show multiple positive foci in papillomas of K14 HPV-8 mice, but not in SCCs of K14 HPV-8/Rac1-EKO mice (Figure 5D–5F)). Such foci have been associated with senescent cells [32, 33]. This makes it conceivable that the absence of Rac1 prevents the maintenance of senescence in UV light damaged keratinocytes, thereby releasing their growth arrest and enabling progression of carcinogenesis. A similar mechanism could explain why, upon constitutive activation of Rac1, K14 HPV-8/K14 L61Rac1 mice develop papillomas, but not carcinomas: in these mice Rac1 dependent mechanisms controlling senescence are still functioning.

Finally, in view of the proven role of Rac1 in the maintenance of the epidermal stem cell compartment [18, 34], the fact that the loss of its function facilitates skin carcinogenesis seems particularly surprising. In contrast to Tiam1 knockout mice, which do not show hair loss, Rac1-EKO mice lose most of their hair-coat within few weeks after birth [18]. This is due to the destruction of the non-permanent part of the hair follicle which harbors the epidermal stem cell niche [34]. There is evidence that SCCs of the skin arise from epidermal stem cells which, in intact hair follicles, reside in the bulge area [35]. An alternative explanation for the occurrence of SCCs in K14 HPV-8/Rac1-EKO mice would therefore be that the loss of Rac1 disrupts the integrity of the epidermal stem cell niche, which normally protects from UV-light induced skin tumor formation [35]. We have observed that Rac1-EKO mice have a smaller bulge area with reduced numbers of keratin 15- and CD34 positive epidermal stem cells (own unpublished observations). We could therefore imagine that the loss of Rac1 leads to an exodus of stem cells from the bulge area into other niches where they are less protected from the mutagenic UV-light and therefore are more prone to the accumulation of persisting DNA mutations.

In summary, we have identified a dual role of Rac1 in HPV-8- and UV-light induced skin carcinogenesis: Rac1, on one hand, transmits growth factor signals which stimulate the growth of papillomas; on the other, its absence facilitates the development of squamous cell carcinomas, probably through the loss of its tissue integrity- maintaining function in the epidermis and hair follicle.

## MATERIALS AND METHODS

### Mouse models used in this study

All animal experiments were conducted in accordance with European, national and institutional guidelines and were approved by local governmental authorities. The following mouse lines were used: Mice expressing the complete early region of the HPV-8 genome under the control of the K14 promoter (K14 HPV-8 mice) [16]; mice transgenic for an inhibitory (K14 N17Rac1) [17] or a constitutively active (K14 L61Rac1) mutant of Rac1 [19], both expressed under the control of the K14 promoter; mice with epidermis specific deletion of Rac1 (Rac1-EKO) [18]. K14 HPV-8/K14 N17Rac1 double transgenic mice and their control K14 HPV-8 mice were used in the C57/Bl6 background. All other mice were used in the FVB/N background backcrossed for at least five generations. Experimental groups of mice were matched for the background as well as age and sex.

### Single dose UV-irradiation experiments

K14 HPV-8 transgenic mice and K14 HPV-8/K14 N17Rac1 double transgenic mice were irradiated as described [20]. Briefly, mice were anaesthetized and the back area (approximately 4 cm^2^) was shaved. Mice were irradiated with single dose of UVA (10 J/cm^2^) and UVB (1 J/cm^2^).

### Chronic UV-irradiation experiments

K14 HPV-8 mice and K14 HPV-8/Rac1-EKO mice were irradiated repeatedly with UVB light using a protocol described previously [21]. Briefly, mice were irradiated three times in a week with a gradually increasing dose under anaesthesia with 2.5% Isofluoran. Mice were shaved before the start of the experiment and at regular intervals thereafter. Mice received a UVB dose of 0.23 J/cm^2^ for the first 12 treatments, 0.41 J/cm^2^ for treatments 13–36, 0.51 J/cm^2^ for treatments 37–48 and 0.61 J/cm^2^ from treatment 49 till the end of the experiment. On average, each mouse received a cumulative dose of > 20 J/cm^2^ UVB.

Mice were monitored during each treatment for their general condition, changes in skin morphology and tumor formation. Time to tumor development was taken until a visible nodule in the irradiation field appeared. The UV-irradiation treatment was stopped for mice with severe skin erosions and their controls. Mice were photographed typically once in a week to monitor the skin changes.

### Histopathology and immunostaining

Tumors and skin samples were excised and tissues were fixed in 4% formalin and subsequently embedded in paraffin. H/E stained tissue sections were obtained using standard procedures. Histopathological examination and immunostainings were carried out as described previously [17]. For γH2AX immunostainings we used primary antibody (# 9718) from Cell signaling technology, USA.

### Quantification of γH2AX positive nuclei and fluorescence intensity

The quantification of γH2AX positive nuclei and fluorescence intensity was carried out using image analysis program (ImageJ, NIH, USA). Percentage number of γH2AX positive cells was determined by counting the total number of DAPI positive cells. At least 1500 cells were analyzed per mouse from K14 HPV-8 and K14 HPV-8/Rac1-EKO mice. Similarly, fluorescence intensities for γH2AX were measured using ImageJ and were normalized to DAPI intensities of tumor samples from K14 HPV-8 and K14 HPV-8/Rac1-EKO mice.

### Primary mouse keratinocyte isolation and culture

Primary epidermal keratinocytes from new-born mice P0-P3 were isolated. Briefly, mice were decapitated, decontaminated by serial washes in povidone-iodine solution, sterile PBS, 70% EtOH and antibiotic-antimycotic solution (Gibco, Life Technologies, Carlsbad, CA, USA) (No. 15240-096) for 30 sec each. Further, whole skin samples were collected from the torso and incubated with 5 mg/ml dispase II (Sigma-Aldrich, St. Louis, MO, USA) (No. D4693-1G) in keratinocyte culture medium overnight at 4°C. The epidermis was separated and was incubated with TrypLE (Gibco, Life Technologies, Carlsbad, CA, USA) (No. 12604-013) for 30 minutes at RT. Afterwards, basal keratinocytes were isolated from the epidermis by several washes with low calcium FAD medium [36], and cultured on collagen type-1 coated cell culture plates in a cell culture incubator at 34°C and 5% CO_2_.

### Western blotting

Skin tissue or cultured keratinocytes were lysed with lysis buffer (1% SDS, 10 mM EDTA). Extracted proteins were denatured at 100°C for 5 min and subjected to electrophoresis using 4–12% Bis-Tris gels (Novex, Life Technologies, Carlsbad, CA, USA) (No. BG04125BOX). Gels were blotted onto polyvinyldiene fluoride membranes using the iBlot transfer system (Invitrogen, Life Technologies, Carlsbad, CA, USA). Afterwards, membranes were incubated in blocking solution (Roche Applied Science, Indianapolis, IN) (No. 11921673001) 1:10 in Tris buffered saline with 0.01% Tween 20 (TBST). Blots were probed with primary antibodies against Myc-tag 1:1000 (Cell Signalling Technology, Danvers, MA, USA) (No. 2276), Rac1 1:500 (Merck Millipore, Schwalbach, Hessen, Germany) (clone 23A8; No. 05-389) and GAPDH 1:5000 in TBST (Trevigen, Gaithersburg, MD, USA) (No. 2275). HRP linked anti-rabbit (No. 7074) or anti-mouse (No. 7076) secondary antibodies (Cell Signalling Technology, Danvers, MA, USA) 1:2000 were used. Immunoreactive proteins were detected using chemiluminescence (PerkinElmer, Waltham, MA, USA) (No. NEL103001EA) and the blots were developed using x ray films (Thermo scientific, Life Technologies, Carlsbad, CA, USA) (No. 34089).
